# Post-traumatic stress disorder symptoms following psychedelic use: a naturalistic survey study

**DOI:** 10.1017/S0033291726103754

**Published:** 2026-04-01

**Authors:** Ricarda Evens, Abdo Uyar, Emily Gosslau, Franziska Dambeck, Dimitris Repantis, Max Wolff, Ulrike Lueken

**Affiliations:** 1 Department of Psychology, Humboldt-Universität zu Berlin, Berlin, Germany; 2 Department of Psychiatry and Neurosciences, CCM, Charité – Universitätsmedizin Berlin, Corporate Member of Freie Universität Berlin and Humboldt-Universität zu Berlin, Berlin, Germany; 3 German Center for Mental Health (DZPG), partner site Berlin-Potsdam, Berlin, Germany

**Keywords:** adverse drug effects, flashbacks, hallucinogenes, psychedelics, PTSD

## Abstract

**Background:**

While clinical research on psychedelics often reports mild and transient side effects, broader survey studies indicate that a subset of users experiences lasting adverse mental health effects. This study investigated whether some of these meet diagnostic criteria for post-traumatic stress disorder (PTSD).

**Methods:**

A cross-sectional online survey (*N* = 243) was conducted with individuals reporting distressing psychedelic experiences with effects persisting beyond the acute phase (convenience sampling). It assessed characteristics of the acute experience, post-traumatic stress, post-traumatic growth, and coping strategies.

**Results:**

A total of 31.3% of participants met the DSM-5 criteria for PTSD as measured by self-report measures. PTSD symptom severity was strongly associated with characteristics of the acute experience. Avoidance-related experiences significantly predicted greater PTSD symptoms, while acceptance-related experiences were linked to lower symptom severity. Post-traumatic growth was unrelated to the intensity of the challenging experience or avoidance but positively predicted by acceptance-related experiences. Post-psychedelic help-seeking behavior was common: most consulted online resources or spoke with friends and family, though psychotherapy was rated the most helpful intervention.

**Discussion:**

Findings provide the first systematic evidence that difficult psychedelic experiences can be associated with later PTSD symptoms and highlight the critical role of acute psychological processes in shaping long-term outcomes. Since the survey targeted individuals with highly challenging acute experiences, the data do not allow the extrapolation of prevalence estimates to the broader population of psychedelic users. As psychedelic use expands beyond clinical settings, access to trauma-informed care and targeted integration support will be essential to minimize harm and support recovery.

## Introduction

Over the past 20 years, research on psychedelics has increased significantly (Uyar, Forbrich, Lueken, & Evens, 2025). The focus of this resurgence has mainly been the investigation of potential clinical benefits in controlled environments. Data on long-term mental health adverse effects from clinical trials conducted in such settings – where there is psychological support and careful screening of patients for risk factors – generally point to a favorable risk profile of psychedelics, although the assessment quality of adverse effects has previously been discussed critically (Breeksema et al., 2022; Hinkle, Graziosi, Nayak, & Yaden, 2024; Romeo et al., 2024; Schlag et al., 2022). Uncontrolled settings, however, may bring factors into play that potentially increase the risk for acute and post-acute difficulties, including lack of preparation, high doses, unsafe settings, unawareness of preexisting vulnerabilities or risk factors, or the combination of different drugs (Evans et al., 2023; Goldy et al., 2025; Marrocu et al., 2024; Nayak et al., 2021; Simonsson et al., 2023). Given that epidemiological studies suggest recreational use of psychedelics is on the rise (Keyes & Patrick, 2023), it is likely that the absolute number of psychedelic-associated mental health complications will increase in the coming years. A comprehensive understanding of these difficulties, as well as potential treatment approaches, is therefore of great importance.

Recent surveys have shown that characteristics of the acute psychedelics experience, especially psychologically difficult or challenging experiences, can be associated with adverse mental health effects (Bouso et al., 2022; Carbonaro et al., 2016; Evans et al., 2023; Simonsson et al., 2023). However, the intensity and duration of these adverse mental health effects are highly heterogeneous. For many individuals, difficulties resolve within days or weeks. Yet across surveys, a small but consistent subset of users reported enduring adverse mental health effects that may persist for months or even years. Participants described a wide range of symptoms, including anxiety and fear, depressive states, social withdrawal, depersonalization, derealization, existential distress, nightmares, and sleep disturbances. A qualitative case analysis based on 15 in-depth interviews with individuals experiencing long-term negative psychological responses to psychedelics further highlighted a predominance of anxiety symptoms, alongside intrusive and obsessive thoughts and general psychological distress (Bremler et al., 2023).

While these studies have been crucial for capturing the range of possible adverse mental health effects associated with psychedelics, they have primarily focused on assessing a broad set of psychopathological symptoms without employing a classificatory diagnostic approach. This work seeks to build on those findings by concentrating specifically on adverse mental health effects that may persist following particularly challenging or traumatic psychedelic experiences. We propose that a subset of these enduring difficulties after psychedelic use are not only transient adjustment symptoms but also meet the full diagnostic criteria for post-traumatic stress disorder (PTSD).

The DSM-5 defines PTSD as a condition marked by a constellation of symptoms that follow a traumatic experience. This event must involve (threat of) death, serious injury, or sexual violence. We hypothesize that psychedelics potentially contribute to the development of PTSD through at least three pathways: (1) extremely challenging or distressing acute psychedelic experiences, (2) the (re-)emergence of traumatic memories during the experience, or (3) traumatic events occurring while under the influence of psychedelics, such as sexual violence or accidents.

Acute psychedelic experiences can be highly challenging, often involving intense emotions such as the fear of death, heightened anxiety, or the feeling of losing one’s mind. Intense emotional overwhelm, perceived loss of control, confrontation with existential fear, or the sudden dissolution of identity and reality boundaries can mirror the subjective characteristics of trauma (Calder, Diehl, & Hasler, 2025). During these experiences, the user’s ability to detach from distressing perceptions may be impaired (Bienemann et al., 2020). Additionally, altered time perception during the experience may exacerbate the sense of fear, as users often perceive time as passing more slowly, which can make the experience feel inescapable (Wittmann et al., 2007).

Furthermore, psychedelic experiences may evoke autobiographical memories of physical or sexual abuse, potentially leading to the development of post-traumatic stress – not in response to the psychedelic experience itself, but to the resurfaced memory (Calder et al., 2025). Notably, some individuals report that these memories had not been previously recalled (Peck et al., 2025; Rose, 2024; Rubin-Kahana, Hassan, & Le Foll, 2021). Because such recollections under the influence of the psychedelic substance often feel subjectively real and are indistinguishable from other memories, they may generate considerable uncertainty about one’s personal history and contribute to significant psychological distress (Aixalà, 2022).

Lastly, it is also possible for a traumatic event to occur while under the influence of a psychedelic substance. Altered states of consciousness can leave individuals in highly vulnerable states, which, under unfavorable circumstances, can be exploited by others. Notably, there have been reports of inappropriate sexual contact by psychedelic sitters, guides, or practitioners (Kruger et al., 2025). However, other external events may also occur during such altered states, including arrests, accidents, or various forms of violence.

Complex interactions between the drug’s effects and the experience of trauma are conceivable. The acute effects may either amplify or diminish the perceived sense of threat, which can influence how the experience is encoded in memory and ultimately affect the likelihood of developing PTSD.

Although many individuals are able to process and manage such experiences without enduring adverse mental health effects, others may develop persistent symptoms of post-traumatic stress. According to the DSM-5-TR, a diagnosis of PTSD requires symptoms across four domains: intrusive memories of the traumatic event; avoidance of trauma-related cues; negative alterations in mood and cognition - such as persistent fear, guilt, or negative beliefs; and hyperarousal, which may manifest as irritability, sleep disturbances, or heightened vigilance (American Psychiatric Association, 2022). These symptoms must persist for more than 4 weeks and lead to clinically significant distress or impairment in social, occupational, or other important areas of functioning.

While many of these symptoms have already been documented at the group level in previous surveys (Calder et al., 2025), no study to date has systematically investigated whether a subset of users with enduring adverse mental health effects meets all diagnostic criteria for PTSD at the individual level. Based on these considerations, the aim of the present study was to address the following research questions:

1. Do certain enduring adverse mental health effects following the use of psychedelics meet the full diagnostic criteria for PTSD?
2. If so, what exactly qualifies as the ‘traumatic event’ in these contexts?
3. How do the qualities of the acute psychedelic experience relate to the symptoms reported afterward?
4. What strategies do individuals use to cope with symptoms, and which approaches are effective?

As previous reports have suggested that even highly challenging psychedelic experiences are sometimes perceived as beneficial in the long term (Peck et al., 2025), this study also examined the potential for post-traumatic growth after such difficult psychedelic experiences, a concept that refers to positive psychological changes that can emerge following highly challenging or traumatic life events (Tedeschi & Calhoun, 2004).

## Methods

### Study procedure

Data were collected through the ‘Study on Traumatic Experiences Related to Psychedelics’, a cross-sectional online survey conducted in both English and German. The survey was available from April 8, 2024, to November 15, 2024. The study received ethical approval from the Ethics Committee of the Institute of Psychology at Humboldt-Universität zu Berlin (Germany) (2024-07R1).

### Inclusion and exclusion criteria

Eligible participants were required to: (1) consent to participate in the study; (2) be at least 18 years old; (3) have used a psychedelic substance at least once; and (4) have experienced a particularly challenging or traumatic psychedelic experience, the aftermath of which continued to affect them even after the acute effects of the substance had subsided. The survey covered classical psychedelics (including psilocybin, LSD, ayahuasca, mescaline, DMT, and 5-MeO-DMT) as well as atypical psychedelics such as MDMA, ketamine, and cannabis. As the term ‘psychedelics’ is not used consistently in the literature, the recruitment materials explicitly stated that participants were eligible to take part in the survey if they had used either typical or atypical psychedelics (‘You have taken a psychedelic substance at least once in your life (e.g. classic psychedelics like psilocybin/Magic Mushrooms, LSD, Ayahuasca, Mescaline, DMT, 5-MeO-DMT, or atypical psychedelics like MDMA/Ecstasy, Ketamine, or Cannabis)’).

### Recruitment

Participants were recruited via social media, online forums, newsletters, and interest groups of psychedelics, employing a convenience sampling method. Information on the survey was provided on a project landing page, which linked to the survey hosted via SoSci Survey (Leiner, 2024). If participants omitted questions from the survey, they were asked to answer the missing questions at the end of each page.

### Study measures

The following section of the survey was used for the present analysis:

#### Demographics

Participants were asked about age, sex, country of residence, and level of education (using the Comparative Analysis of Social Mobility in Industrial Nations (CASMIN) classification of education (Brauns, Scherer, & Steinmann, 2003).

#### Description of index experience

Participants were instructed to respond to all questions with reference to the particularly challenging or traumatic psychedelic experience they reported at study inclusion. If they had experienced multiple such episodes, they were asked to focus on the one that had caused the greatest difficulties in the period following the experience.

*Descriptives.* Participants were asked to report the time elapsed since the index experience (ordinal from ‘less than 6 months’ to ‘more than 20 years’), the clarity of their memory (five-point Likert-type scale from ‘not clear at all’ to ‘completely clear’, the specific psychedelic substance used (multiple choice list with substances), the perceived intensity of the dose (five-point Likert-type scale from ‘low’ to ‘extremely high’) and its acute effects (five-point Likert-type scale from ‘very weak’ to ‘very strong’), the duration (ordinal from ‘less than 1 h’ to ‘more than 10 h’) and emotional valence of those effects (categorical, see Table 3), how challenging the experience was (four-point Likert-type scale from ‘not challenging at all’ to ‘very challenging’), and their retrospective evaluation of it (five-point Likert-type scale from ‘very negative’ to ‘to very positive’). Additional information was collected on concomitant substance use, prior psychedelic experiences, and psychedelic use following the index experience (multiple-choice list with substances). For specific labels of all intermediate levels, see Table 3.

*Setting.* Participants were asked several dichotomous (yes/no) questions referring to specific setting categories (calm outdoor; calm indoor; designed and/or prepared for a therapeutic objective; party, concert or festival; psychedelic retreat; ceremonial, religious or spiritual; research), number of people present during the experience, whether a support person was available, their level of familiarity with that person, and whether the support person was also under the influence of a psychoactive substance.

*Use motives.* Use motives were assessed as previously described (Wolff et al., 2022, 2024). Participants were presented with a list of 22 motives for using psychedelics (e.g. ‘out of curiosity’, ‘to treat psychological problems’, and ‘to have fun’), and were asked to rate the extent to which each item corresponded to their motives for undergoing the reported experiences at that time on a four-point Likert-type scale (‘not at all’, ‘somewhat’, ‘moderately’, and ‘very much’).

*Characterization of the index experience.* Participants were asked which of the following descriptions most accurately reflected their experience during the index event: (1) encountering challenging or frightening thoughts, perceptions, or feelings; (2) recalling challenging or traumatic experiences from their past – participants were explicitly encouraged to respond even if they were uncertain whether these memories were grounded in actual events; and (3) experiencing a threatening or dangerous event (e.g. assault, accident, abuse, or rape). If participants reported more than one of these experiences, they were asked to indicate which aspect had affected them the most in the days and weeks following the experience.

Participants who either recalled past memories or experienced a threatening event during the index experience were asked to identify the nature of the event using the standardized Life Events Checklist for DSM-5 (LEC-5), with the option to report events not included in the checklist.

Those in the *memory group* were asked to indicate their age at the time the remembered event occurred, whether they had any memory of the event prior to the psychedelic experience, and, if so, whether they had been bothered by the memory before the index experience. Additionally, they were asked to rate their confidence in the memory being based on a real event using a visual analogue scale (VAS) ranging from ‘not at all sure’ to ‘very sure’.

Participants in the *event group* were asked whether they believed the psychedelic substance had altered their perception of potential danger or threat, and how dangerous or threatening they considered the situation in retrospect.

The following questionnaires were used to characterize the acute experiences:

*Challenging Experience Questionnaire (CEQ).* The CEQ (Barrett et al., 2016; Dworatzyk, Jansen, & Schmidt, 2022) consists of 26 items assessing various dimensions of psychologically difficult experiences during the acute effects of psychedelics, including fear, grief, physical distress, insanity, isolation, death, and paranoia. In the present study, the total score was used for analysis.

*Acceptance/Avoidance-Promoting Experiences Questionnaire (APEQ).* The APEQ (Wolff et al., 2022) consists of 32-items that assess acceptance- and avoidance-related psychedelic experiences. In the present analysis, the two main scales, Acceptance (comprising the subscales Accepting Response, Relief, and Acceptance-Related Insights, APEQ-ACE) and Avoidance (comprising the subscales Avoidant Response, Distress, and Avoidance-Related Insights, APEQ-AVE) were used for analysis.

#### Post-traumatic stress and post-traumatic growth

*Post-traumatic stress.* PTSD symptoms were assessed using the PTSD Checklist for DSM-5 (PCL-5) (Blevins et al., 2015; Krüger-Gottschalk et al., 2017). The PCL-5 is a 20-item questionnaire that measures the severity of PTSD symptoms based on the DSM-5 diagnostic criteria. Each item corresponds to one of the core symptom clusters – intrusion, avoidance, negative alterations in cognition and mood, and hyperarousal – and is rated on a five-point Likert scale ranging from 0 (not at all) to 4 (extremely). For the purposes of this study, the standard instruction was slightly modified: instead of referring to symptoms over the past month, participants were asked to rate their symptoms with reference to the month they found most challenging following their psychedelic experience. While the PCL-5 is a well-validated screening tool that captures symptoms across all four DSM-5 symptom clusters, it does not assess the diagnostic criteria F (duration >1 month) and G (clinically significant distress or impairment). Therefore, participants were additionally asked how long disturbances persisted and whether these disturbances caused significant distress or impairment in social, occupational, or other important areas of functioning. Furthermore, participants were asked about how much they were currently suffering from the disturbances using a visual analogue scale ranging from ‘not at all’ to ‘very much’. In an open-text field, participants were also invited to describe any persistent symptoms or difficulties they experienced in the days, weeks, months, or even years following the index experience.

*Post-traumatic growth.* Post-traumatic growth was measured using the Post-Traumatic Growth Inventory (PTGI) (Maercker & Langner, 2001; Tedeschi & Calhoun, 1996). The PTGI consists of 21 items that assess various dimensions of positive psychological change following trauma, including relating to others, new possibilities, personal strength, spiritual change, and appreciation of life. In the present study, the total score was used for analysis.

*Diagnostics.* Participants were asked whether they had ever been diagnosed with a mental disorder specifically related to symptoms following the difficult psychedelic experience, as well as whether they had ever received any other mental health diagnoses during their lifetime.

#### Therapeutic attempts

Participants were asked about any attempts they had made to address their symptoms. They rated the 10 items shown in Figure 4 using the response options: *Not tried, Not helpful at all, Somewhat helpful, and Very helpful.* Additionally, participants were invited to provide further details in an open-text field, describing any other attempts they had made.

### Analysis

Data quality was assessed, and participants were excluded if they showed indicators of poor data quality: (a) a relative speed index above the recommended cut-off of 2.0 (Leiner, 2019) combined with no engagement in free-text comments, or (b) free-text responses that indicated a lack of genuine participation. This included statements that clearly indicated that the participant was not completing the questionnaire (e.g. ‘just looking’) or did not answer the questions (e.g. insults). This review process was conducted by two reviewers.

Participants were assigned to the PTSD group if they met all of the following criteria: (a) endorsement of symptoms in all four symptom clusters on the PCL-5, (b) a total PCL-5 score exceeding the clinical cut-off of 32, (c) symptom duration of more than 1 month, and (d) significant distress or impairment in social, occupational, or other important areas of functioning caused by disturbances related to the index experience. Participants who did not meet all of these criteria were assigned to the non-PTSD group. It is important to note that group assignment into the PTSD and non-PTSD groups was not based on a clinical interview, which represents the gold standard for diagnosing PTSD. Instead, the fulfillment of diagnostic criteria was based solely on participants’ self-reports.

Differences in demographics, acute, and post-acute symptoms were described and compared between the groups.

Principal component analysis (PCA) with orthogonal Varimax rotation was used to reduce the dimensionality of the 22 assessed use motives and to capture the most important use motive patterns in the data. Component scores were computed using the regression method. Items with negative loadings were retained, indicating that higher endorsement corresponds to lower values on the component. No item reversals were performed. Higher component scores reflect greater alignment with the pattern of loadings, and individual item contributions should be interpreted according to their loading signs.

Linear regression models were used to predict PCL-5 and PTGI scores from the CEQ. Multiple regression models were employed to predict PCL-5 and PTGI scores from the APEQ-AVE, APEQ-ACE, and their interaction (using standardized scores).

Statistical analyses were performed using IBM SPSS Statistics (Version 29) and R (Version 4.4.3). Statistical significance was set at *p* < .05.

## Results

### Sample description

A total of 1,195 volunteers began the survey, and 512 provided informed consent to participate. Of these, 269 participants were excluded for the following reasons: not meeting inclusion criteria (*n* = 90), discontinuation of the survey before completing the PCL-5 items (*n* = 176), free-text responses indicating a lack of genuine participation (e.g. ‘just reading’; *n* = 2), or exceeding the recommended relative speed index cut-off (> 2) without any indication of engagement in free-text comments (*n* = 1). The final sample consisted of 243 participants. Sample characteristics are presented in Table 1.

**Table 1.** Sample characteristics

	Total sample		PTSD		Non-PTSD		df	*t* or *χ* ^2^	p	Effect size^a^
	N	%	N	%	N	%				
	243	100%	76	31.3%	167	68.7%				
Age (mean, SD)	37.93	(14.01)	34.37	(12.09)	39.57	(14.56)	173	2.906	.004	0.376
Sex							3	1.174	.759	0.070
Female	90	37.0%	31	40.8%	59	35.3%				
Male	139	57.2%	40	52.6%	99	59.3%				
Non-Binary	12	4.9%	4	5.3%	8	4.8%				
Other	2	0.8%	1	1.3%	1	0.6%				
Language							1	1.785	.182	0.086
English	148	60.9%	51	67.1%	97	58.1%				
German	95	39.1%	25	32.9%	70	41.9%				
Country							34	24.633	.881	0.318
USA	46	18.9%	19	25.0%	27	16.2%				
UK	17	7.0%	6	7.9%	11	6.6%				
Germany	97	39.9%	27	35.5%	70	41.9%				
Switzerland	10	4.1%	4	5.3%	6	3.6%				
Other	73	30.0%	20	26.3%	53	31.7%				
Education (CASMIN)							2	6.766	.034	0.167
Primary education	11	4.5%	3	3.9%	8	4.8%				
Secondary education	44	18.1%	24	27.6%	23	13.8%				
Tertiary education	188	77.4%	52	68.3%	136	81.4%				
Mental Health Diagnoses										
Before Index Exp.	139	57.2%	50	65.8%	89	53.3%	1	2.507	.113	0.103
After Index Exp.^b^	52	21.4%	38	50.0%	14	8.6%	1	51.821	<.001	0.467

*Note*:

a Cohen’s *d* or Cramer’s *V.*

b Mental health disorder due to the symptoms following the difficult psychedelic experience.

### Levels of post-traumatic stress after the index experience

**Figure 1.**
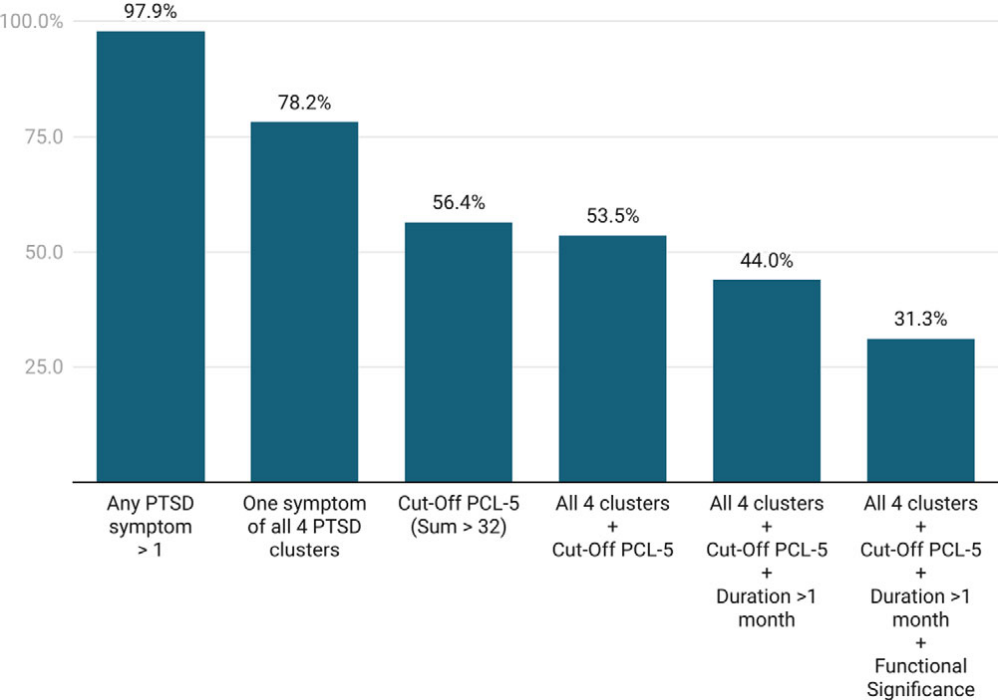
Levels of post-traumatic stress after the index experience.

Nearly all participants reported at least one PTSD symptom as measured by the PCL-5; 31.3% of participants reported symptoms from all four PTSD symptom clusters, met the PCL-5 cut-off of 32, reported a symptom duration of more than 1 month, and indicated that these disturbances caused significant distress or impairment in their social, occupational, or other important areas of functioning, and were therefore classified as part of the PTSD group. Figure 1 illustrates all intermediate screening steps for identifying PTSD symptoms. The mean intensity of PTSD symptoms in the PTSD group was 55.63, which is well above the recommended clinical PCL-5 cut-off score of 32. In terms of demographics, the PTSD group was significantly younger and had a lower level of education than the non-PTSD group (see Table 1). Although there was no statistically significant difference in the frequency of mental health diagnoses between the groups prior to the index experience, a significantly higher proportion of participants in the PTSD group reported being diagnosed with a mental health disorder as a result of symptoms following the difficult psychedelic experience (see Table 1).

Regarding the post-psychedelic symptoms, the PTSD group showed a higher PCL-5 score in all four symptom clusters and longer symptom durations. By definition, all participants of the PTSD group reported significant distress or impairment in their social, occupational, or other important areas of functioning. Participants in the PTSD group also reported greater current psychological distress compared to the non-PTSD group. Although not statistically significant, there was a trend toward lower levels of posttraumatic growth in the PTSD group. All test statistics are shown in Table 2.

**Table 2.** Acute and post-acute symptoms of the index experience

	Total sample		PTSD		Non-PTSD		df	*t* or *χ* ^2^	*p*	Effect size^a^
ACUTE										
CEQ	0.54	(0.21)	0.64	(0.19)	0.49	(0.20)	241	−5.43	<.001	0.752
APEQ										
Avoidance-related experience	61.43	(21.96)	69.48	(20.06)	57.77	(21.86)	241	−3.969	<.001	0.547
Acceptance-related experience	32.25	(26.33)	25.25	(22.78)	35.43	(27.26)	172	3.031	.003	0.392
Introspection	75.04	(23.03)	75.35	(24.93)	74.90	(22.19)	241	−0.140	.889	0.019
Interaction	49.56	(25.59)	45.39	(25.11)	51.46	(25.19)	241	1.721	.087	0.238
POST-ACUTE										
PTSD symptoms (PCL–5)	35.93	(19.97)	55.63	(10.86)	26.96	(16.42)	210	−16.109	<.001	1.922
Intrusions	9.62	(5.96)	14.33	(4.48)	7.48	(5.28)	169	−10.431	<.001	5.045
Avoidance	3.67	(2.69)	5.46	(2.10)	2.86	(2.53)	173	−8.392	<.001	2.403
Mood and Cognitions	13.64	(7.63)	20.45	(4.10)	10.54	(6.81)	223	−14.032	<.001	6.094
Arousal	9.00	(6.81)	15.40	(4.41)	6.09	(5.62)	182	−13.950	<.001	5.272
Duration of PTSD disturbances							7	76.042	<.001	0.559
No persisting disturbances	30	12.3%	0	0.0%	30	18.0%				
1–3 days	16	6.6%	0	0.0%	16	9.6%				
4–7 days	14	5.8%	0	0.0%	14	8.4%				
1–4 weeks	29	11.9%	0	0.0%	29	17.4%				
1–6 months	54	22.2%	18	23.7%	36	21.6%				
7–12 months	38	15.6%	19	25.0%	19	11.4%				
1–5 years	38	15.6%	25	32.9%	13	7.8%				
>5 years	24	9.9%	14	18.4%	10	6.0%				
Distress or Impairment	109	51.2%	76	100%	33	19.8%	1	112.740	<.001	0.728
Current suffering (VAS 0–100)^b^	33.92	33.21	46.26	(34.92)	27.08	(30.29)	137	−4.022	<.001	0.599
Posttraumatic Growth (PTGI)^c^	45.87	29.84	41.16	(27.82)	48.11	(30.58)	228	1.656	.099	0.234

*Note*: The table shows means and standard deviations, while ‘duration of PTSD symptoms’ and ‘distress or impairment’ are presented as frequencies and percentages.

a Cohen’s *d* or Cramer’s *V.*

b In participants that reported any persistent disturbances.

c Sample size *n* = 230 (non-PTSD group *n* = 156; PTSD group *n* = 74).

### Description of the index experience

Details of the index experience are presented in Table 3. Most participants described an index experience involving either LSD or psilocybin. Approximately one-third reported experiences with more than one substance, with 49 participants (56.3%) indicating a combination of LSD, psilocybin, or MDMA with cannabis. Aside from alcohol, concurrent use of non-psychedelic substances was rare. The majority of participants (82.7%) reported that their index experience had occurred more than 6 months prior. Only a few described the experience as involving a low dose of psychedelics; most reported the subjective intensity of the acute effects as strong or very strong. More than half of the participants (65.8%) reported that the index experience took place in a calm indoor setting, and 31.7% indicated that the setting had been designed and/or prepared for a therapeutic objective.

**Table 3.** Characteristics of psychedelic index experience

	Total sample		PTSD		Non-PTSD		df	*t* or χ^2^	*p*	Effect size^a^
	*N*	*%*	*N*	*%*	*N*	*%*				
	243	100%	76	31.3%	167	68.7%				
Index substance							11	9.640	.563	0.199
LSD	50	20.6%	14	18.4%	36	21.6%				
Psilocybin	46	18.9%	17	22.4%	29	17.4%				
DMT	2	0.8%	0	0.0%	2	1.2%				
5-MeO-DMT	2	0.8%	1	1.3%	1	0.6%				
Ayahuasca	22	9.1%	4	5.3%	18	10.8%				
Mescaline	2	0.8%	0	0.0%	2	1.2%				
DOM or DOI	0	0.0%	0	0.0%	0	0.0%				
2C-B	3	1.2%	0	0.0%	3	1.8%				
MDMA	5	2.1%	2	2.6%	3	1.8%				
Cannabis	9	3.7%	5	6.6%	4	2.4%				
Ketamine	11	4.5%	4	5.3%	7	4.2%				
Other psychedelics	4	1.6%	2	2.6%	2	1.2%				
I do not know	0	0.0%	0	0.0%	0	0.0%				
Multiple substances from list	87	35.8%	27	35.5%	60	35.9%				
Concomitant drug use										
None	186	76.5%	50	65.8%	136	81.4%	1	7.123	.008	0.171
Alcohol	35	14.4%	14	18.4%	21	12.6%	1	1.448	.229	0.077
Amphetamines	7	2.9%	2	2.6%	5	3.0%	1	0.025	.876	0.010
Cocaine	8	3.3%	2	2.6%	6	3.6%	1	0.152	.697	0.025
Opiates	4	1.6%	3	3.9%	1	0.6%	1	3.617	.057	0.122
Benzodiazepines	4	1.6%	0	0.0%	4	2.4%	1	1.851	.174	0.087
Other dissociatives	6	2.5%	3	3.9%	3	1.8%	1	1.003	.316	0.064
Inhalants	1	0.4%	1	1.3%	0	0.0%	1	2.206	.137	0.095
Others	14	5.8%	9	11.8%	5	3.0%	1	7.531	.006	0.176
Date of use							5	5.982	.308	0.157
Less than 6 months ago	42	17.3%	7	9.2%	35	21.0%				
6–12 months	38	15.6%	11	14.5%	27	16.2%				
1–5 years	94	38.7%	32	42.1%	62	37.1%				
6–10 years	28	11.5%	11	14.5%	17	10.2%				
11–20 years	14	5.8%	5	6.6%	9	5.4%				
More than 20 years ago	27	11.1%	10	13.2%	17	10.2%				
Dosage							4	2.325	.676	0.098
Low	4	1.6%	1	1.3%	3	1.8%				
Moderate	68	28.0%	22	28.9%	46	27.5%				
High	93	38.3%	27	35.5%	66	39.5%				
Very high	50	20.6%	14	18.4%	36	21.6%				
Extremely high	28	11.5%	12	15.8%	16	9.6%				
Duration of acute effects							4	3.236	.519	0.115
Less than 1 h	14	5.8%	4	5.3%	10	6.0%				
1–2 h	17	7.0%	4	5.3%	13	7.8%				
3–5 h	66	27.2%	17	22.4%	49	29.3%				
6–10 h	93	38.3%	35	46.1%	58	34.7%				
>10 h	53	21.8%	16	21.1%	37	22.2%				
Subjective intensity of acute effects							3	0.281	.963	0.034
Weak	5	2.1%	2	2.6%	3	1.8%				
Moderate	38	15.6%	11	14.5%	27	16.2%				
Strong	80	32.9%	25	32.9%	55	32.9%				
Very strong	120	49.4%	38	50.0%	82	49.1%				
Clarity of memory							4	2.406	.662	0.100
Not clear at all	1	0.4%	0	0.0%	1	0.6%				
Somewhat clear	33	13.6%	11	14.5%	22	13.2%				
Clear	75	30.9%	19	25.0%	56	33.5%				
Very clear	98	40.3%	33	43.4%	65	38.9%				
Completely clear	36	14.8%	13	17.1%	23	13.8%				
Rating of acute effects							3	5.178	.159	0.146
Rather pleasant	10	4.1%	2	2.6%	8	4.8%				
Rather unpleasant	118	48.6%	45	59.2%	73	43.7%				
Both pleasant and unpleasant	108	44.4%	27	35.5%	81	48.5%				
Neither pleasant, nor unpleasant	7	2.9%	2	2.6%	5	3.0%				
Acute challenges^b^							3	2.670	.445	0.105
Not challenging at all	1	0.4%	0	0%	1	0.6%				
Slightly challenging	10	4.1%	3	3.9%	7	4.2%				
Moderately challenging	59	24.3%	14	18.4%	45	26.9%				
Very challenging	173	71.2%	59	77.6%	114	68.3%				
Retrospective evaluation of experience^c^							4	19.102	<.001	0.280
Very negative	76	31.3%	36	47.4%	40	24.0%				
Negative	53	21.8%	19	25.0%	34	20.4%				
Neither negative nor positive	47	19.3%	10	13.2%	37	22.2%				
Positive	42	17.3%	8	10.5%	34	20.4%				
Very positive	25	10.3%	3	3.9%	22	13.2%				
Frequency of psychedelic use BEFORE							4	8.180	.085	0.183
Never	36	14.8%	18	23.7%	18	10.8%				
1–5 times	77	31.7%	22	28.9%	55	32.9%				
6–20 times	68	28.0%	19	25.0%	49	29.3%				
21–50 times	33	13.6%	7	9.2%	26	15.6%				
>50 times	29	11.9%	10	13.2%	19	11.4%				
Frequency of psychedelic use AFTER							4	3.860	.425	0.126
Not at all	65	26.7%	26	34.2%	39	23.4%				
1–5 times	82	33.7%	23	30.3%	59	35.3%				
6–20 times	56	23.0%	14	18.4%	42	25.1%				
21–50 times	23	9.5%	7	9.2%	16	9.6%				
>50 times	17	7.0%	6	7.9%	11	6.6%				
Setting										
Calm outdoor setting (e.g. nature)	57	23.5%	17	22.4%	40	24.0%	1	0.073	.787	0.017
Calm indoor setting (e.g. apartment)	160	65.8%	49	64.5%	111	65.5%	1	0.092	.761	0.019
Party, concert, or festival	50	20.6%	17	22.4%	33	19.8%	1	0.217	.641	0.030
Psychedelic Retreat	38	15.6%	9	11.8%	29	17.4%	1	1.208	.272	0.070
Ceremonial, religious, or spiritual event	25	10.3%	4	5.3%	21	12.6%	1	3.025	.082	0.112
Clinical research setting	11	4.5%	4	5.3%	7	4.2%	1	0.139	.710	0.024
Setting designed and/or prepared for a therapeutic objective	77	31.7%	25	32.9%	52	31.1%	1	0.074	.785	0.018
Number of people present							6	1.741	.942	0.085
0 (alone)	46	18.9%	16	21.1%	30	18.0%				
1 other person	66	27.2%	20	26.3%	46	27.5%				
2–5 people	55	22.6%	15	19.7%	40	24.0%				
6–15 people	29	11.9%	11	14.5%	18	10.8%				
16–30 people	16	6.6%	4	5.3%	12	7.2%				
31–100 people	11	4.5%	4	5.3%	7	4.2%				
>100 people	20	8.2%	6	7.9%	14	8.4%				
Sitter present	107	44.0%	35	46.1%	72	43.1%	1	0.183	.669	0.027
Sitter under influence?	55	22.6%	15	19.7%	40	24.0%	1	1.520	.218	0.119
Sitter known							3	1.638	.651	0.124
Not at all	10	4.1%	4	5.3%	6	3.6%				
Somewhat	19	7.8%	5	6.6%	14	8.4%				
Moderately	27	11.1%	11	14.5%	16	9.6%				
Very much	51	21.0%	15	19.7%	36	21.6%				

*Note*:

a Cramer’s V.

b ‘How challenging was the acute experience?’

c ‘Looking back, how do you evaluate the experience you had by taking the substance(s)?’

The PTSD and non-PTSD groups differed significantly in terms of concomitant drug use, with the PTSD group reporting more concurrent substance use than the non-PTSD group. Participants in the PTSD group also retrospectively evaluated the index experience as more negative than those in the non-PTSD group. Although not statistically significant, there was a trend suggesting that a greater proportion of individuals in the PTSD group had no prior experience with psychedelics compared to the non-PTSD group (23.7% versus 10.8%). Additionally, there was a trend toward a higher percentage of participants in the non-PTSD group reporting that the index experience took place in a ceremonial, religious, or spiritual setting compared to the PTSD group (12.6% versus 5.3%).

*Use motives.* Testing of the prerequisites indicated that the use motives were suitable for a PCA: Bartlett’s test of sphericity was significant, *χ* ^2^(231) = 2,251.724, *p* < .001, and the Kaiser–Meyer–Olkin (KMO) measure of sampling adequacy was 0.812. Inspection of the anti-image correlation matrix further supported this suitability. PCA revealed five components with eigenvalues greater than 1; however, the scree plot suggested a three-component solution as more appropriate. These three components cumulatively explained 49.2% of the total variance. Based on the component loadings presented in Table 4, the components were labeled (1) hedonic intention, (2) self-exploration intention, and (3) escapist intention.

**Table 4.** Item loadings from the principal component analysis (PCA) of use motives in the complete bilingual sample (*N* = 243)

	Component 1: ‘Hedonic intention’	Component 2: ‘Self-Exploration’	Component 3: ‘Escapist intention’
To treat psychological problems	**−0.779**	0.034	0.322
To confront difficult feelings	**−0.738**	0.067	0.175
To have fun	**0.727**	0.001	0.446
To spend time with friends	**0.713**	0.007	0.072
For partying	**0.600**	−0.218	0.208
To intoxicate myself	**0.590**	−0.106	0.497
To fit in	**0.487**	−0.090	0.085
Out of curiosity	**0.448**	0.305	0.197
To increase my well-being	**−0.422**	0.371	0.325
To treat physical problems	**−0.363**	0.037	0.282
To increase my creativity	0.057	**0.731**	0.255
To have an experience of nature	0.179	**0.728**	0.110
For spiritual reasons	−0.265	**0.713**	−0.122
For self-awareness	−0.491	**0.666**	−0.184
For personal growth	−0.561	**0.627**	−0.170
For religious reasons	0.011	**0.529**	−0.150
To increase my performance	−0.281	**0.452**	0.160
To distract myself from problems	0.086	−0.041	**0.759**
To escape from difficult feelings	−0.222	−0.037	**0.712**
Out of boredom	0.270	−0.048	**0.537**
For relaxation	0.196	0.419	**0.496**
To increase sexual pleasure	−0.014	0.060	**0.460**

*Note*: Items were rated on a four-point Likert-type scale (‘not at all’, ‘somewhat’, ‘moderately’, and ‘very much’).

The highest loading of each item is written in bold font.

There was no significant difference between the PTSD and non-PTSD groups in hedonic intentions, *t*(241) = 0.605, *p* = .546, *d* = 0.084. However, participants with PTSD symptoms reported significantly stronger escapist intentions, *t*(241) = 2.164, *p* = .031, *d* = 0.299, and weaker self-exploration intentions, *t*(241) = −3.445, *p* < .001, *d* = −0.477.

**Figure 2.**
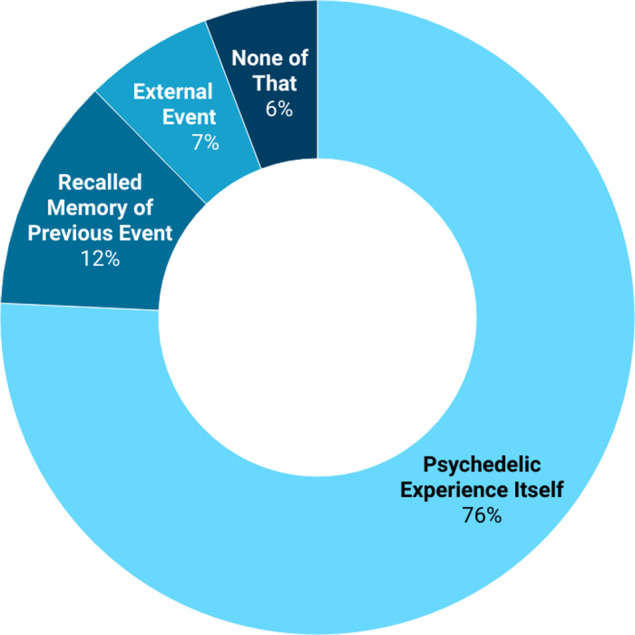
Description of index experience. *Note*: The figure shows responses to the question: ‘Which aspects of your experience have affected you the most in the days and weeks following the experience?’ *N* = 243 (all study participants), responses only within the PTSD group were similar: experience 76%, memory 13%, event 8%, none 3%.

*Acute experiences.* When asked which of the following descriptions most accurately reflected their psychedelic index experience (multiple responses allowed), 215 participants (88.5%) reported encountering very challenging or frightening thoughts, perceptions, or feelings; 74 participants (30.5%) recalled challenging or traumatic experiences from their past; 27 (11.1%) reported experiencing a threatening or dangerous event during the acute drug effect (e.g. assault, accident, abuse, or rape), and 14 (5.8%) stated that none of the descriptions applied to their experience. Seventy-nine participants (32.5%) indicated that more than one of these descriptions matched their experience. Figure 2 displays the responses to the follow-up question regarding which aspect of the experience had most affected them in the days and weeks following the psychedelic experience (forced single choice). There was no statistically significant difference between the PTSD and non-PTSD groups in this regard (*χ* ^2^(3) = 2.313, *p* = .510, *V* = 0.098).

While all participants described their acute experiences as very challenging in the CEQ and reported more avoidance-related than acceptance-related experiences on the APEQ, these ratings were significantly more pronounced in the PTSD group compared to the non-PTSD group (see Table 2).

*Memories.* In the subgroup that indicated memories had affected them the most in the days and weeks following the psychedelic experience, the following types of memories – based on the Life Events Checklist for DSM-5 (LEC-5) – were reported: 11 participants (37.9%) recalled sexual assault; one (3.4%) other unwanted or uncomfortable sexual experience; two participants each (6.9%) recalled a life-threatening illness or injury, and sudden violent death (e.g. homicide or suicide); and one participant each (3.4%) reported memories of a fire or explosion or severe human suffering. In nine cases (31.0%), the category ‘any other very stressful event or experience’ was selected. These included: feeling poisoned, organized and ritual abuse, psychological abuse, death of a mother, infidelity, issues with an ex-partner, heartbreak and personal traumas, fear of punishment, and negative childhood memories.

Regarding memory status prior to the psychedelic experience, 13 participants (44.8%) reported having no prior memory of the event, 14 (48.3%) had prior memories, and two (6.9%) were unsure. Among those who had prior memories or were unsure, 12.5% indicated the memory had not bothered them at all before, 25.0% were somewhat bothered, 25.0% were moderately bothered, and 37.5% were very much bothered by the memory before the psychedelic experience.

When asked how confident they were that the memory was based on real events (on a visual analog scale from 0 to 100), participants with prior memories reported significantly higher confidence (mean = 97.07, SD = 7.71) compared to those without prior memory (mean = 65.69, SD = 42.02), *t*(13) = 2.651, *p* = .020.

On average, participants reported that the recalled event occurred at the age of 11.38 years (SD = 9.45, median = 8.00 years). In 41.4% of cases, the reported age was 6 or younger, and in 72.4% of cases, the age was 12 or younger. The age of the event was significantly lower in the group without prior memories (mean = 6.38 years, median = 4.00 years) compared to the group with prior memories (mean = 16.71 years, median = 15.00 years), *t*(20) = 3.298, *p* = .002.

*Event.* Among participants who identified a threatening or dangerous event during their psychedelic experience as the most impactful in the days and weeks afterward, the following memories from the LEC-5 were reported: 2 cases (12.5%) of physical assault, one case each (6.3%) of transportation accident, sexual assault, and life-threatening illness or injury. In 11 cases (68.8%) the category ‘any other very stressful event or experience’ was chosen, including being arrested and sent to jail, contact with police, difficult/threatening contact with people also under the influence of a substance or with people being psychotic, mobbing, unconsented actions by practitioner, blackout and having to be constrained by friends, being caught in a cult. One quarter of the participants indicated that their perception of threat was unchanged by the substance use, 31.3% reported reduced, and 43.8% an increased perception of danger. In retrospect, 12.5% indicated that the situation was not at all, 18.8% somewhat, 31.3% moderate, or 37.5% very dangerous.

*Experience reports.* The following examples from the open-text field on persisting symptoms illustrate participants’ subjective experiences of post-psychedelic effects:

‘It’s now been 3 months since my retreat. I continue to have constant anxiety, with consistent flashbacks. My anxiety shows up as: claustrophobia (I can’t wear necklaces or tight clothing), an inability to breath (like something is sitting on my chest), overheating, a feeling of panic, constant intrusive thoughts. I will flashback and “be back in my journey” 1-3 times a week. I’m having fewer nightmares, but I do still have them’.

‘I suffer from severe anxiety and derealisation/depersonalisation/dissociation. Several times a day, for the past 7 years I feel as if I am again experiencing the ayahuasca ceremony. I feel as if I am in a different dimension, away from everybody around me. It happens mostly when I think about the event, or when I hear music, smells, or conversations about ayahuasca of different dimensions etc. It seriously impacts my life and I can’t do the things in my life that I wish to do’.

‘I had horrible flashbacks, I cried a lot and felt very distanced from people. I was easily triggered into tears if something happened in my family or my partner. I re-experienced the experience and kept wondering why I had to go through that, I could not make sense of it all, and even though I took support and talked openly with my partner and friends the flashbacks kept happening and I felt really horrible’.

‘panic attacks, social anxiety, dissociation and derealization, nightmares, existential confusion about my identity and meaning of life, depression at loss of social abilities, fatalism, suicidal thinking, fear of permanent damage, substance abuse (alcohol) to mask symptoms’

‘[…] In the two months after recovering the memory, I experienced the following: near-constant flashbacks, traumatic nightmares, the recovery of countless other traumatic memories […], nausea/vomiting related to shame, restlessness and insomnia, panic attacks, paranoia, an impending sense of doom, random bouts of crying, random bouts of euphoria / spiritual openness, and constant terror. I ended up going to in-patient treatment for two months, which, in many ways, saved my life’.

#### Prediction of post-traumatic stress and post-traumatic growth

**Figure 3.**
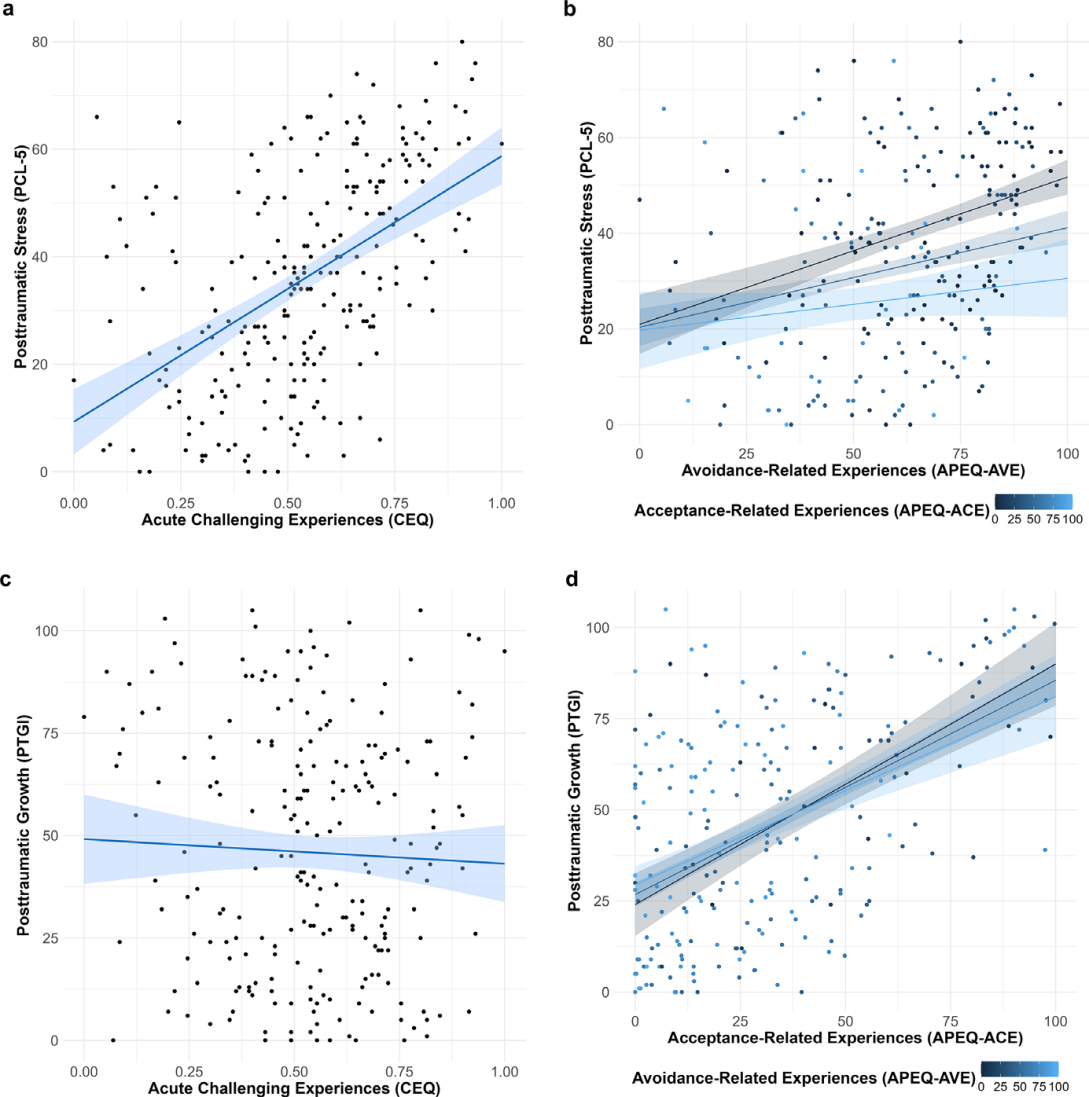
Prediction of posttraumatic stress and posttraumatic growth. *Note*: The plot displays unstandardized scores for both predictor and outcome variables. Upper panels illustrate associations between posttraumatic stress and (a) acute challenging experiences and (b) avoidance- and acceptance-related experiences of the index event. Lower panels illustrate associations between posttraumatic growth and (c) acute challenging experiences and (d) avoidance- and acceptance-related experiences of the index event.

*Post-traumatic stress.* The linear regression model predicting PCL-5 scores from the CEQ was significant, *F*(1, 241) = 86.051, *R* ^2^ = .263, *p* < .001, indicating a positive association between acute challenging experiences and post-traumatic stress symptoms (*t* = 9.276, *β* = .513; Figure 3a).

The multiple regression model predicting the PCL-5 total score from APEQ-AVE, APEQ-ACE, and their interaction was also significant, *F*(3, 239) = 12.004, *p* < .001, *R* ^2^ = .131: Both APEQ-AVE (*t* = 4.123, *β* = .268, *p* < .001) and APEQ-ACE (*t* = −2.639, *β* = −.178, *p* = .009) were significant, whereas their interaction was not (*t* = −0.985, *β* = −.058, *p* = .325). Avoidance-related experiences during the index experience were associated with higher levels of post-traumatic stress symptoms, while acceptance-related experiences were associated with lower symptom levels (Figure 3b).

*Post-traumatic growth.* The linear regression model predicting PTGI scores from the CEQ was not significant, *F*(1, 228) = 0.398, *R* ^2^ = .002, *p* = .529, indicating no association between acute challenging experiences and post-traumatic growth (*t* = −0.631, *β* = −.042; Figure 3c).

In contrast, the multiple regression model predicting PTGI scores from APEQ-AVE, APEQ-ACE, and their interaction was significant, *F*(3, 226) = 26.805, *p* < .001, *R* ^2^ = .262: APEQ-ACE (*t* = 7.986, *β* = .504, *p* < .001,) but neither APEQ-AVE (*t* = 0.103, *β* = .006, *p* = .918,) nor the interaction term (*t* = −.510, *β* = −.028, *p* = .611) were significant predictors. Acceptance-related experiences during the index experience were associated with more post-traumatic growth (Figure 3d).

#### Therapeutic attempts

**Figure 4.**
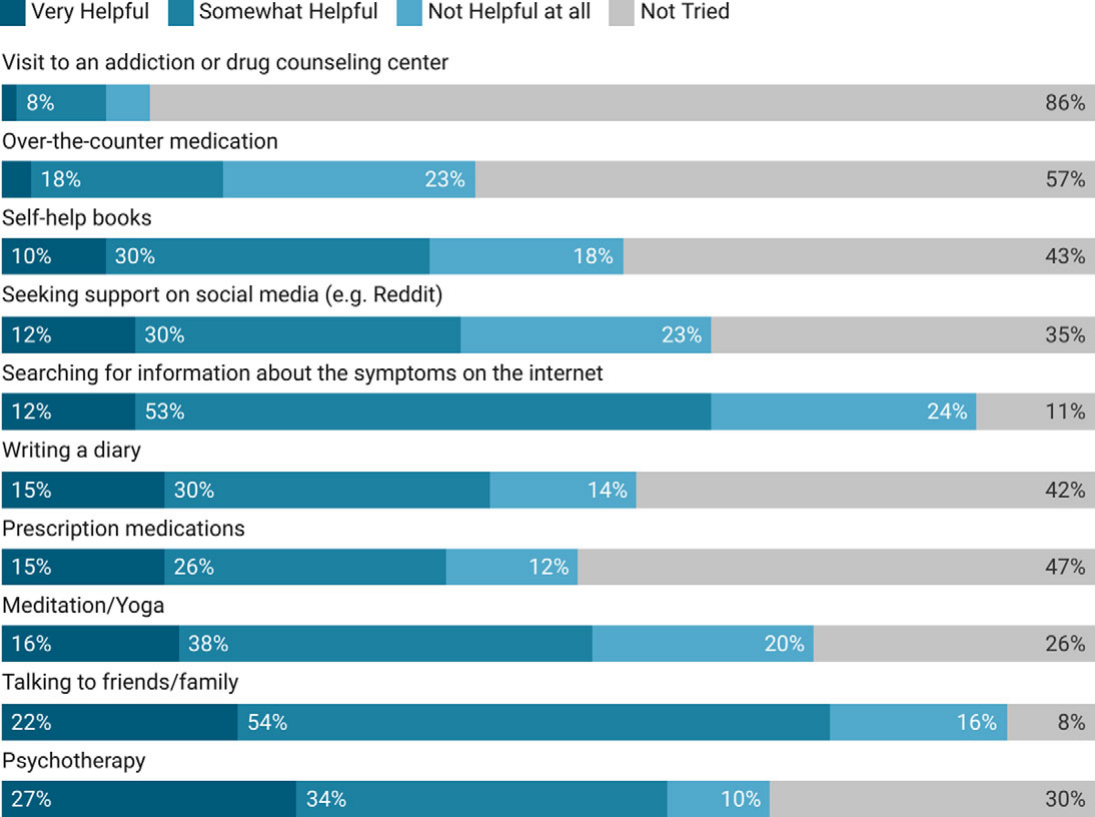
Therapeutic attempts. *Note*: The figure shows responses to the question: ‘Have you ever tried any of the following therapy/supportive measures for your problems, and how have they helped you?’ Data based on *n* = 74 participants of PTSD group.

The majority of participants (*n* = 158, 65.0%) reported having made efforts to address problems that emerged following their psychedelic experience. A significantly higher proportion of individuals in the PTSD group reported such efforts compared to the non-PTSD group (85.1% versus 59.7%), *χ^2^*(1) = 14.910, *p* < .001, *V* = 0.253. Figure 4 illustrates the therapeutic approaches reported by participants in the PTSD group. Additionally, 29 participants in the PTSD group (38.2%) reported engaging in other forms of therapy or supportive measures, encompassing a broad range of approaches such as inpatient treatment, acupuncture, breathwork, re-engaging with psychedelics, hypnotherapy, ice bathing, physical exercise, electroconvulsive therapy (ECT), and specific forms of psychotherapy (e.g. EMDR or CPT).

## Discussion

This study investigated whether certain enduring adverse mental health effects after challenging or traumatic psychedelic experiences can be associated with symptoms of post-traumatic stress disorder (PTSD). Among 243 individuals who reported having had such an experience followed by lasting difficulties, 31.3% met the full diagnostic criteria for PTSD as measured by self-report measures. Most participants (76%) reported being primarily affected by very challenging or frightening thoughts, perceptions, or feelings encountered during the experience; 12% reported recalling traumatic or distressing events from their past, and 7% indicated that a threatening or dangerous event occurred while they were under the influence of psychedelics. The severity of PTSD symptoms was strongly associated with the intensity of the acute challenging experience. High levels of avoidance-related experiences and low levels of acceptance-related experiences were linked to greater post-traumatic stress. At the same time, many participants also reported elements of post-traumatic growth. While this growth was not associated with how challenging the index experience was, nor with the extent of avoidance-related experiences during the acute phase, it was positively predicted by the degree of acceptance-related experiences reported during the acute psychedelic experience. Approximately two-thirds of the participants sought information about their symptoms online, and 92% engaged in conversations with friends and family. However, psychotherapy was rated as the most helpful therapeutic approach.

### Can certain post-psychedelic challenges be understood as a form of PTSD?

The findings of this study clearly demonstrate that challenging or traumatic psychedelic experiences can be associated with the development of full PTSD symptomatology. In our sample, 31.3% of participants met the full diagnostic criteria for PTSD as measured by self-report measures. While this proportion may appear high at first glance, it is important to note that the study specifically targeted individuals who reported having had at least one particularly difficult or traumatic psychedelic experience with persisting aftereffects after the acute effects of the substance had subsided. A recent epidemiological study conducted in an unselected sample of *classic* psychedelic users found that 3.4% reported challenging or distressing effects lasting longer than 1 month (Simonsson et al., 2023). In our sample, among those who experienced any PTSD symptoms for more than 1 month, 49.4% (all psychedelics) and 47.7% (classic psychedelics) reported symptoms from all four PTSD symptom clusters, met the PCL-5 cut-off of 32, and indicated that these disturbances caused significant distress or impairment in their social, occupational, or other important areas of functioning. Extrapolating roughly from the epidemiological data above, this suggests a very approximate prevalence of 1.7% (all psychedelics) and 1.6% (classic psychedelics) of PTSD cases among the broader population of psychedelic users. However, this should be considered as an upper-bound estimate, as the 3.4% reporting prolonged challenging or distressing effects cited above likely include persistent mental health problems other than PTSD, such as depressive disorders.

The course of symptoms was frequently chronic: 33% reported symptom durations between 1 and 5 years, and 18% reported durations exceeding 5 years. These results directly contradict common claims in the field, suggesting that the adverse effects of psychedelics are invariably mild and transient, and instead call for a more nuanced consideration of risks and adverse outcomes and the need for harm reduction approaches.

*Risk factors.* Demographically, participants were on average middle-aged, though the PTSD group was slightly younger and showed a statistical trend toward a higher likelihood of the index experience being their first psychedelic use (24% versus 11%). While the overall sample was relatively well-educated, participants in the PTSD group had lower average levels of education. A high rate of pre-existing lifetime mental health diagnoses was found in the full sample (57%), with the PTSD group showing numerically higher – but not statistically significantly different – rates (66%). The most commonly reported substances used during the index experience were LSD and psilocybin, which are consistent with the fact that these are the most commonly used serotonergic psychedelic substances (Lake & Lucas, 2024). However, no significant differences emerged between the PTSD and non-PTSD groups regarding the type of psychedelic substances used. Non-classical psychedelics such as MDMA and cannabis were reported at comparatively low rates, especially when contrasted with their overall prevalence of use. This finding is in line with a previous study on extended difficulties after psychedelic use, which also reported comparatively low rates of such difficulties following the use of cannabis, MDMA, and ketamine (Evans et al., 2023). One possible explanation is that these substances are less likely to induce intensely challenging acute experiences than classic serotonergic psychedelics and, consequently, may be associated with fewer cases of PTSD (Strickland, Garcia-Romeu, & Johnson, 2024). In the case of MDMA, it has even been suggested that its therapeutic potential partly stems from its ability to allow individuals to confront difficult emotions without becoming overwhelmed – an effect summarized under the term ‘helioscope hypothesis’ (Hasler, 2022).

Notably, participants in the PTSD group were significantly more likely to have combined psychedelics with other, non-psychedelic substances during the index experience (34.2% versus 18.6%), suggesting that polydrug use may represent a potential risk factor for the development of PTSD symptoms following the use of psychedelics.

While contextual factors are frequently cited as crucial in shaping post-psychedelic outcomes, the present data did not reveal significant differences in setting variables between the PTSD and non-PTSD groups. Many PTSD cases occurred despite the presence of commonly assumed protective elements: participants often reported calm environments, and nearly half indicated that a sitter had been present. A non-significant trend suggested that ceremonial, religious, or spiritual contexts might be associated with a lower incidence of subsequent PTSD. It is plausible that highly ritualized settings may not only buffer against acutely overwhelming experiences but also offer a more structured framework for post-experience processing through shared meaning-making practices and communal support.

However, differences emerged in relation to participants’ motivation for substance use: individuals in the PTSD group were more likely to report escapist motives and less likely to endorse self-exploratory intentions. This pattern aligns with existing literature identifying emotional avoidance as a risk factor for the development of PTSD (Orcutt, Reffi, & Ellis, 2020), as well as with findings on the differential psychological outcomes of avoidance- versus approach-related motives in psychedelic use (Soto-Angona et al., 2025; Wolff et al., 2022, 2024), highlighting the potentially crucial role of preparation and intention-setting in the clinical application of psychedelics to maximize outcomes and support harm reduction.

### What characterizes psychedelic-related traumatic experiences?

If PTSD symptoms related to psychedelic substance use are a possible outcome, the question arises as to what qualifies as the index trauma in these cases. A large proportion of participants described their acute experiences as being marked by intensely challenging or frightening thoughts, perceptions, or emotions, which they directly associated with the emergence of post-psychedelic symptoms. This connection is further supported by a strong association between the intensity of acutely challenging psychedelic experiences and the severity of subsequent PTSD symptoms.

Notably, 12% of participants attributed their distress primarily to the resurfacing of difficult autobiographical memories, particularly of childhood sexual abuse. A substantial subset of these individuals reported that they had no prior recollection of such events before the psychedelic experience. This group expressed wide variability in their subjective certainty regarding the veracity of these memories, with a moderate level of confidence on average. Among those with newly recalled memories, the average age at the time of the remembered event was significantly younger than among those who recalled already-known events (6 versus 17 years). Within the latter group, there was variation in the degree of prior distress: some individuals had already been burdened by these memories, while others had not. These findings suggest that psychedelics may not only reframe or alleviate the emotional impact of past traumatic events – as observed in MDMA-assisted psychotherapy or in ceremonial retreat contexts (Feduccia & Mithoefer, 2018; Weiss, Wingert, Erritzoe, & Campbell, 2023) – but may also amplify or elicit new distressing responses to such memories. While therapeutic settings with psychedelics may intentionally facilitate emotional processing and integration of trauma, spontaneous and unprepared resurfacing of traumatic memories during psychedelic use without therapeutic support may contribute to the onset of PTSD symptoms, especially in individuals with strong avoidant coping tendencies. Furthermore, the findings of the present study also align with previous research reporting associations between difficulties following naturalistic psychedelic use and adverse childhood experiences, although the underlying causal mechanism of this association has yet to be established (Olofsson et al., 2026).

A particularly sensitive and controversial issue concerns the question of false memories. The emergence of previously unknown memories under the influence of psychedelics raises the possibility that some recollections may not correspond to actual past events. For affected individuals, questioning the autobiographical basis of such memories can feel deeply invalidating and may be perceived as a denial of their lived reality. However, from both clinical and ethical standpoints, this topic demands careful and transparent discussion. Unquestioned acceptance of potentially false memories can have serious consequences for individuals’ psychological well-being, sense of identity, and social relationships. While a recent study suggested that the psychedelic state may offer some protection against the formation of false memories, this finding specifically pertains to the pre-encoding administration of psychedelics (Doss et al., 2024). In contrast, certain features of the psychedelic state – such as heightened suggestibility and emotional intensity – may actually increase vulnerability to false memory formation (Healy, 2021). This highlights the urgent need for further research on the relationship between psychedelics and memory processes (Kangaslampi & Lietz, 2025). Clinical guidelines for addressing ‘recovered’ memories usually recommend that clinicians maintain a stance of supportive neutrality, avoiding assumptions about whether such memories are accurate or inaccurate (Courtois, 2002). Therapeutic work may focus on helping individuals cope with ambiguity and build greater tolerance for uncertainty. Even when specific memories cannot be verified, the associated emotional experiences can remain clinically significant and may be effectively addressed using interventions common in trauma-focused therapy – such as identifying and modifying unhelpful beliefs about oneself, others, or the world that may have been shaped by the memory (Evens, Uyar, & Majić, 2025).

In addition to internal or memory-related factors, a smaller subset of participants attributed their post-psychedelic distress to external events that occurred during the experience. Interestingly, not all of them were typical events that would meet conventional trauma criteria as defined by the DSM-5 (e.g. serious injury, sexual violence, or threatened death, as listed in the LEC-5). Instead, participants described events such as encounters with law enforcement or frightening and chaotic interactions with other intoxicated individuals. While these incidents may not constitute formal traumatic events per se, their psychological impact must be considered within the altered perceptual and emotional state induced by psychedelics. In fact, 44% of those affected reported heightened threat perception during the experience. This aligns with prior research highlighting the important role of subjective appraisals of an event in the development of PTSD symptoms (Ehlers & Clark, 2000). Thus, the interaction between the pharmacological effects of the substance and situational stressors may create a subjectively traumatic experience, even when the objective event does not meet standard trauma definitions. We argue, however, that a full presentation of PTSD symptomatology following a psychedelic experience should not be dismissed solely because the experience does not meet the formal definition of a traumatic event (DSM-5 Criterion A), but that such experiences are clinically relevant and may warrant trauma-specific support. We are aware, however, that this interpretation may be viewed as controversial, as there is an ongoing debate within PTSD research about whether the ‘gatekeeping function’ of Criterion A is necessary to define a disorder already characterized by a specific set of symptoms (Howard et al., 2024; Kilpatrick, Resnick, & Acierno, 2009; Van Hooff et al., 2009). Furthermore, unlike the DSM-5, the ICD-11 defines trauma more broadly, describing it not in terms of specific situations but as ‘exposure to an extremely threatening or horrific event or series of events’, which could arguably encompass very challenging psychedelic experiences. Nevertheless, because this study focused on DSM-5 criteria – and in the absence of a formal clinical interview – we refer to the PTSD group as exhibiting PTSD symptoms rather than a formal PTSD diagnosis.

It is important to mention that findings from other studies suggest that experiencing a traumatic event under the acute influence of classic psychedelics or MDMA may also reduce the likelihood of post-traumatic stress responses (Karp Barnir et al., 2025; Netzer et al., 2024). Whether the experience of potentially traumatic events under the influence of psychedelics increases or decreases the likelihood of developing PTSD symptoms can, however, not be determined from the present study, as no control group was included. However, the data suggest that for some individuals, PTSD symptoms were closely linked to external events that occurred during the psychedelic experience.

### How do psychological processes during the psychedelic experience relate to the difficulties reported afterward?

Participants in the PTSD group retrospectively described their index psychedelic experience more frequently as ‘very challenging’ compared to the non-PTSD group. Characteristics of the acute experience emerged as strong predictors of subsequent posttraumatic stress symptoms. Specifically, higher levels of perceived challenge, greater avoidance-related experiences, and lower acceptance-related experiences during the acute phase were each associated with greater severity of subsequent PTSD symptoms. These findings are consistent with a previous study that has observed a link between the difficulty of the acute psychedelic experience and duration of post-psychedelic difficulties (Evans et al., 2023).

Interestingly, both the PTSD and non-PTSD groups exhibited quite similar levels of posttraumatic growth, with the PTSD group showing only slightly lower scores. The extent of growth was not associated with how challenging the index experience was perceived to be. Instead, it was significantly related to the degree of acceptance-related experiences during the acute psychedelic state. This finding challenges the common assumption that difficult experiences per se foster psychological growth, and instead suggests that how these challenges are processed in the moment – particularly through acceptance – may play a more critical role in facilitating positive long-term outcomes. A similar pattern was observed in a previous study among ayahuasca users, which likewise found no significant correlation between the intensity of challenging experiences and posttraumatic growth (Cassidy, Healy, Henje, & D’Andrea, 2024).

### What strategies have been used to find relief from symptoms, and which approaches have proven effective?

The severity of post-psychedelic distress in the sample is also reflected in the fact that 85% of individuals in the PTSD group reported having sought some form of treatment to address their difficulties. As expected, commonly used strategies included talking to friends and family, as well as searching for symptom-related information online. While a large majority (89%) of help-seeking participants consulted internet resources, only 12% found them to be very helpful. This suggests that either educational materials alone are insufficient for alleviating symptoms, or that accessible, relevant information on post-psychedelic PTSD is currently lacking online. Moreover, encountering generalized reassurances – such as claims that complications are typically ‘mild and transient’ – may further exacerbate distress and contribute to feelings of isolation among those experiencing more severe or persistent symptoms (Robinson et al., 2024).

Among the strategies reported, psychotherapy was rated as the most helpful intervention. This finding is consistent with established clinical guidelines for the treatment of PTSD, which recommend trauma-focused psychotherapy as the first-line intervention (Hamblen et al., 2019). However, as the survey did not assess which specific modalities of psychotherapy were utilized, it remains unclear whether participants received treatments explicitly designed for PTSD. It is likely that some individuals engaged in more general forms of psychotherapy that may not have directly addressed PTSD symptoms.

### Limitations

This is the first study to systematically examine cases of PTSD symptoms related to psychedelic use. While our findings provide important initial insights, several limitations should be acknowledged.

First, the study was designed to investigate whether PTSD symptoms related to psychedelic use occur at all, rather than to estimate their prevalence. Therefore, we specifically targeted individuals who retrospectively described their experience as highly challenging or traumatic and reported persisting symptoms beyond the acute effects. As a result, while the findings confirm that PTSD symptoms related to psychedelic use do occur, they do not allow the direct extrapolation of prevalence estimates to the broader population of psychedelic users.

Furthermore, this survey included experiences with both classical and atypical psychedelics, although most participants reported using classical psychedelics. Given the limited number of cases, particularly for atypical substances, the present data did not allow for a meaningful differentiation of effects between substance groups, and subgroup analyses according to substance type were therefore not conducted.

As with many survey-based studies, there are potential sources of sampling bias. Similar to other psychedelic surveys, our sample was predominantly composed of male participants from Western countries, with relatively high educational backgrounds and a mean age in mid-adulthood. This limits generalizability, particularly given that trauma prevalence, expression, and appraisal are known to vary considerably across cultural and sociodemographic contexts (Brown, 2008; Ford, 2008; Roberts, Watlington, Nett, & Batten, 2010; Stamm & Friedman, 2000). Of the 422 volunteers who provided informed consent and met the inclusion and exclusion criteria, 243 participants completed all sections relevant to the present analysis, corresponding to a non-completion rate of 42%. Although this rate is not negligible, it is not unexpected given the medium length of the survey, the absence of completion incentives (e.g. financial compensation), and the inclusion of sensitive topics such as substance use and mental health. The individual reasons for survey discontinuation are unknown. However, we do not expect that attrition compromised the interpretability of the main results. Attrition may even have tended to bias the sample toward participants with lower levels of psychological distress, as individuals experiencing higher distress may have been more likely to discontinue the survey due to the burden associated with responding to sensitive questions.

A particular strength of the present study is that, at the individual case level, it assessed not only PTSD symptoms but also the duration of symptoms and the level of associated distress or functional impairment. However, it is important to note that PTSD was evaluated solely using self-report measures. No clinical interviews or comprehensive differential diagnostic procedures were conducted, which limits the diagnostic certainty of the findings. A previous comparison of the self-report measure PCL-5 and the structured clinical interview CAPS-5 in military and veteran treatment-seeking samples has shown that total scores tend to be higher in self-report measures than in clinical interviews (Resick et al., 2023). This suggests that using self-report measures may result in an overestimation of PTSD cases. In the present study, we attempted to mitigate this risk by assessing not only the PCL-5 but also the other diagnostic criteria, particularly symptom duration and functional impairment.

Moreover, because no differential diagnostic procedures were conducted, it cannot be ruled out that some cases identified as meeting PTSD criteria might be more accurately explained by alternative diagnoses (e.g. depressive disorders). Although the data from this study provide the strongest evidence to date for the potential emergence of PTSD in connection with psychedelic use, further research using structured clinical interviews is needed to replicate these findings.

Another focus of the survey was to explore the relationship between aspects of the acute experience and post-acute symptoms. Based on the initial hypothesis that PTSD would be more strongly associated with difficult and avoidant, rather than accepting, acute experiences, we prioritized the inclusion of measures assessing these aspects of the acute experience. However, psychedelic experiences encompass a much broader phenomenology of other perceptual, cognitive, and emotional changes, and the present study does not permit conclusions about how these other effects may relate to post-acute outcomes.

### Future directions

This study provides important initial evidence that cases of PTSD following experiences with psychedelics do exist. However, many questions remain open. One of the most pressing concerns is how to effectively support affected individuals. At present, there is no reason to assume that well-established, evidence-based interventions for PTSD – particularly trauma-focused psychotherapy – would not also be applicable in these cases. While different therapeutic models may emphasize different components, two overarching treatment strategies can be identified: first, structured engagement with the traumatic memory and second, the exploration and reappraisal of thoughts and beliefs shaped by the traumatic experience, which may affect how individuals perceive themselves, others, and the world and that maintain distressing emotions such as fear, guilt, shame, anger, or sadness (Schnyder et al., 2015). Nevertheless, it may still be valuable to systematically identify and document specific cognitive patterns and beliefs that commonly emerge in this context. For example, individuals may report persistent fears of having sustained irreversible psychological damage. Clinicians unfamiliar with psychedelic-related psychopathology may find it challenging to assess the plausibility or evidence of such beliefs.

Another open question is whether psychedelic-assisted psychotherapy could itself be a helpful treatment for PTSD following traumatizing experiences with psychedelics – and if so, through which mechanisms. One rationale is that MDMA-assisted therapy may support processes such as memory reconsolidation and fear extinction related to the traumatizing memory by allowing individuals to confront difficult emotions related to those memories without becoming overwhelmed (Feduccia & Mithoefer, 2018; Hasler, 2022). Alternatively, repeated administration of the same psychedelic substance experienced during the index event – effectively re-exposing the individual to the feared stimulus, namely the psychedelic state itself – could allow for reassessment and re-evaluation of fears associated with altered states of consciousness, potentially enabling new psychedelic experiences that foster inhibitory learning (Craske et al., 2014). However, while these approaches may hold theoretical promise, they would require careful consideration of ethical implications and a thorough assessment of the potential for further harm.

Given the relatively small absolute number of cases and their geographically widespread distribution, a more feasible and accessible approach to supporting affected individuals would be the development and optimization of internet-based information and support resources. This is particularly relevant considering that the majority of affected individuals in our study reported turning to the internet for support.

This study also highlights the potential involvement of autobiographical memory processes during the psychedelic state in the emergence or exacerbation of PTSD symptoms. However, this remains a largely under-explored research area, and many important questions are still unanswered (Kangaslampi & Lietz, 2025). Debates reminiscent of the so-called ‘memory wars’ have begun to reemerge – particularly in relation to concerns that psychedelics might either elevate the risk of false memory formation or facilitate the recovery of previously ‘suppressed’ memories (Dodier, Otgaar, & Mangiulli, 2024; Patihis et al., 2014). However, whereas earlier debates often adopted a dichotomous stance, recent non-psychedelic research has shifted toward a more nuanced perspective that recognizes the complex interplay of cognitive, social, and emotional factors in the resurfacing of long-forgotten trauma. Applying this more sophisticated framework to psychedelic contexts could be crucial for both research and clinical practice moving forward (Dodier et al., 2024).

As noted in the limitations section, socio-demographic and cultural factors are likely to play a significant role in shaping how PTSD symptoms manifest (Stamm & Friedman, 2000). Another important direction for future research should therefore be to investigate how these variables influence the vulnerability to, experience of, and recovery from adverse mental health effects after the use of psychedelics. Such an approach would help uncover culturally specific meanings attributed to psychedelic experiences, as well as variations in coping mechanisms, social support systems, and stigma. A more contextually grounded understanding is essential for developing culturally sensitive therapeutic frameworks and harm-reduction strategies (Bryant-Davis, 2019).

### Conclusion

This study provides the first systematic evidence that challenging or traumatic psychedelic experiences can be associated with later Criterion A symptoms of post-traumatic stress disorder. While the sample is not representative of the general population of psychedelic users, the findings clearly demonstrate that a subset of individuals – particularly those who experience high levels of distress during the acute psychedelic state – may develop clinically significant and often long-lasting adverse mental health effects after the use of psychedelics. Importantly, these outcomes may result not only from the intensity of the psychedelic experience itself, but also from the resurfacing of autobiographical memories or the occurrence of distressing external events under the influence of psychedelics.

The data highlight the critical role of psychological processes during the acute experience – particularly avoidance and acceptance, the constructs assessed in this study – which were associated with both posttraumatic stress and posttraumatic growth. This indicates the potential relevance of fostering acceptance-based coping strategies in the clinical application of psychedelics.

The high rate of help-seeking and the strong endorsement of psychotherapy underscore the importance of ensuring access to trauma-informed care for those affected. Recognizing and addressing the potential for psychedelic-related traumatization is not intended to discredit psychedelic therapy or personal use, but is essential for maintaining a balanced and evidence-based understanding of both the risks and benefits of psychedelic use and therapy.
